# Genomic Analysis of the Insect-Killing Fungus *Beauveria bassiana* JEF-007 as a Biopesticide

**DOI:** 10.1038/s41598-018-30856-1

**Published:** 2018-08-17

**Authors:** Se Jin Lee, Mi Rong Lee, Sihyeon Kim, Jong Cheol Kim, So Eun Park, Dongwei Li, Tae Young Shin, Yu-Shin Nai, Jae Su Kim

**Affiliations:** 1 Department of Agricultural Biology, College of Agriculture & Life Sciences, Chonbuk National University, Jeonju, 54596 Korea; 2 Department of Biotechnology and Animal Science, National Ilan University, Ilan, Taiwan; 3 Plant Medical Research Center, College of Agricultural and Life Sciences, Chonbuk National University, Jenoju, 54596 Korea

## Abstract

Insect-killing fungi have high potential in pest management. A deeper insight into the fungal genes at the whole genome level is necessary to understand the inter-species or intra-species genetic diversity of fungal genes, and to select excellent isolates. In this work, we conducted a whole genome sequencing of *Beauveria bassiana* (*Bb*) JEF-007 and characterized pathogenesis-related features and compared with other isolates including *Bb* ARSEF2860. A large number of *Bb* JEF-007 genes showed high identity with *Bb* ARSEF2860, but some genes showed moderate or low identity. The two *Bb* isolates showed a significant difference in vegetative growth, antibiotic-susceptibility, and virulence against *Tenebrio molitor* larvae. When highly identical genes between the two *Bb* isolates were subjected to real-time PCR, their transcription levels were different, particularly in *heat shock protein 30* (*hsp30*) gene which is related to conidial thermotolerance. In several *B*. *bassiana* isolates, *chitinases* and *trypsin-like protease* genes involved in pathogenesis were highly conserved, but other genes showed noticeable sequence variation within the same species. Given the transcriptional and genetic diversity in *B*. *bassiana*, a selection of virulent isolates with industrial advantages is a pre-requisite, and this genetic approach could support the development of excellent biopesticides with intellectual property protection.

## Introduction

The main goal of pesticides is to control and reduce the population density of target pest insects to below an economic threshold. However, the emergence of environmental toxicity and insect resistance strengthens the regulation of synthetic pesticides^1^. Situations are now encountered where the strategy in pest management is to select between synthetic pesticides and biopesticides. Synthetic pesticides with a novel mode of action could be developed with reduced environmental toxicity, but at the same time, biopesticides with a more efficacious control activity could be developed. Future decisions probably depend on the speed of technological development to resolve these issues. Recently, a strategic collaboration between synthetic pesticides and biopesticides has been generated^2^. However, in the near future, unique biopesticides could be a major asset in pest management due to the development of faster technology.

In pest management, insect-killing fungi (entomopathogenic) have high potential in reducing pest populations in agriculture and forests and even as medical vector controls^3^. Many Ascomycota species such *as Beauveria*, *Metarhizium*, *Lecanicillium*, *Isaria*, and others have been studied, and some isolates have been developed for agriculture and forest pest management^4^. One of the advantages of using a fungal biological control agent in pest management is its broad spectrum, whereby one isolate can cover several species of insects including lepidopteran, orthopteran, thysanopteran, heteropteran, dipteran, and coleopteran insects, compared to the narrow range of other microbial control agents, such as *Bacillus thuringiensis* and insect-pathogenic virus and nematodes^5–7^. Secondly, entomopathogenic fungi can be easily spread and transmitted within insect populations or over different populations in favorable environmental conditions. A closed system such as a polyvinyl greenhouse can be environmentally controlled to allow the fungi to express high virulence against insects, leading to a combination of digital farming and biological control. Successful entomopathogenic fungi-mediated pest management has not been frequently reported, but in the near future, a well-controlled environmental system is going to enlarge the fungal insecticide market.

The entomopathogenic fungal conidia, asexual spores, attach to the cuticle of target insects and germinate. Germination is possibly triggered by signals from insect cuticles and environmental cues such as carbohydrates and a minimum amount of moisture^8–11^. After germination, hyphal penetration of the insect cuticle occurs, where the physical forces of hyphae and enzymatic degradation of the exo- and endo-cuticles plays important roles in this pathogenesis process^12,13^. Such fungal infection processes encounter insect immune responses, such as melanization and cellular pathway-mediated defense^14–16^. After overcoming the insect immune system, infecting fungi finally reach the insect hemocoel, where they use insect nutrition and form blastospores in the liquid state of the insect inner spaces. This consequently results in the death of the target insect and second growth on the surface of the insect cadaver for transmission to other target pests.

Many studies of entomopathogenic fungi have focused on their biological application and physiological response to environmental stresses, but less effort has been devoted to downstream process studies^3,17^. In downstream processing, studies in solid and liquid-based culture systems and formulation should receive more interest for the successful application of entomopathogenic fungi in field conditions. As described above, entomopathogenic fungi have many advantages in pest management, such as a broad host spectrum, environmental soundness, and higher safety to non-target organisms including humans, but they have poor stability during storage and distribution, even after application in field conditions. In addition, a relatively high production cost needs to be considered for successful pest management. Substrate manipulation in solid and liquid cultures and oil-based formulations have now significantly improved fungal thermotolerance and stability^18^. Owing to the development of technology, which is possibly derived from food microbiology-related fermentation, the production costs are approaching acceptable levels. However, still more efforts are required to increase its potential from an industrialized standpoint.

Recently, more attention has been given to not only industrial-related technology, but also to the study of entomopathogenic fungi pathogenesis (mode of action). A clearer understanding of fungal action in pathogenesis will allow more effective and efficient application methodology to be established. Several novel findings, such as factors on conidial adhesion, cuticle degradation, nutrient assimilation, and stress management during fungal pathogenesis, have been reported in this area of research^14^. In *B*. *bassiana* and *Metarhizium*, adhesion-related genes^19–22^, cuticle-degradation genes^23,24^, stress management-related genes^25,26^, adaption to insect immune response-related genes^27,28^, multifunctional transcription factors^29,30^, and nutrient assimilation-related genes^31^ have been studied. Studies on the interactions between insects and entomopathogenic fungi have focused on the comparative transcriptome^32–34^.

Further insight of fungal genes at the whole genome level is necessary to understand the role of genes in pathogenesis and the genetic diversity of fungal genes in interspecies or intra-species. Additionally, in the same species, for example within the same *B*. *bassiana* species, genetic diversity could explain diverse biological performance among the isolates. In this work, we conducted a whole genome sequencing of *B*. *bassiana* JEF-007^34–36^ using PacBio RS sequencing and further investigated some important genes in pathogenesis. Particularly, the genome of *B*. *bassiana* JEF-007 was compared to other well-known entomopathogenic fungi, such as another *B*. *bassiana* isolate and the two species *Cordyceps* and *Metarhizium*. The JEF-007 genome was further analyzed by comparison with the ARSEF2860 strain of the same species. JEF-007 genes with a high identity to ARSEF2860 genes were studied to compare the gene expression between two different isolates. Additionally, JEF-007 genes with a low identity to ARSEF2860 were further compared with other *B*. *bassiana* isolates (about 6 or 10) including GHA (BotaniGard^TM^, Bioworks, USA) and ERL836 (Chongchaesak^TM^, LG-Chemical-affiliated FarmHannong, Korea) to analyze the sequence variation (diversity) of *B*. *bassiana* genes.

## Results

### Pathogenicity of *Bb* JEF-007 against insect pests

**Figure 1:**
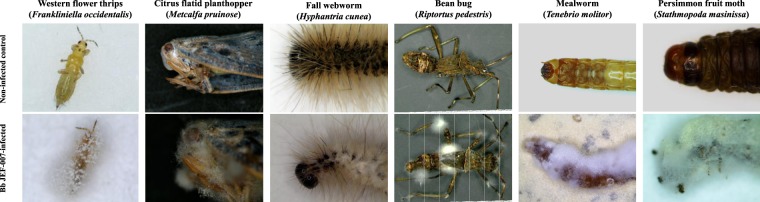
Insecticidal activity (virulence) of *B*. *bassiana* JEF-007 against western flower thrips, citrus flatid planthopper, fall webworm, bean bug, mealworm and persimmon fruit moth in laboratory conditions. The JEF-007 conidial suspension (1 × 10^7^ conidia/ml) was sprayed on the adult, nymph or larva at 2 ml/dish, and the dishes were kept at 25 ± 1 °C (60% RH) for 7~10 days. The above photos were taken by the authors of this article.

In our bioassays, *Bb* JEF-007 showed pathogenicity against important agricultural and forest insect pests, western flower thrips, citrus flatid planthopper, fall webworm, bean bug, mealworm and persimmon fruit moth (Fig. 1). Particularly in 5 days after the inoculation, active sporulation was observed on the thrips which had resistance against several chemical insecticides and needs alternative control agents. Hyphal growth and conidiation was observed on forest insects, fall webworm and persimmon fruit moth larvae.

### Whole genome sequencing of *Bb* JEF-007

The concentration of *Bb* JEF-007 gDNA in the PacBio sequencing was 88.484 ng/ul, and a total of 10.176 ug of gDNA was used for the sequencing (Supplementary Fig. S1). The PacBio sequencing of JEF-007 generated ca. 5.46 Gbp of nucleotides, and the average subread length was 8,471 bp. A total of 645,008 reads were sequenced with an N_50_ of ca. 12.025 kb. After the *de novo* assembly, 39 contigs were produced, and the genome size of JEF-007 was predicted to be 36.358 Mb with an N_50_ of 3.1 kb (Supplementary Table S1).

### Annotation of *Bb* JEF-007 genes

**Figure 2:**
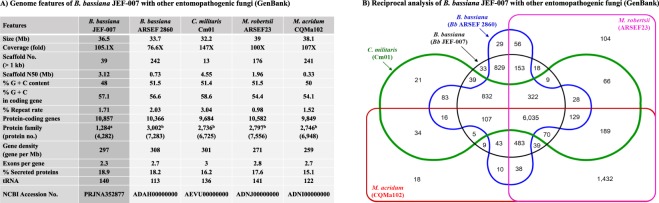
Genome features of *B*. *bassiana* JEF-007 and other entomopathogenic fungi (**A**) and reciprocal analysis of the five major entomopathogenic fungi, *B*. *bassiana* JEF-007 and ARSEF2860, *C*. *militaris* Cm01, *M*. *robertsii* ARSEF23 and *M*. *acridum* CQMa102 (**B**).

When the overall genomic features of JEF-007 (GenBank accession number: PRJNA352877) were compared with other entomopathogenic fungi, the JEF-007 genome size of 36.4 Mb was similar to *Bb* ARSEF2860 (33.7 Mb), *C*. *militaris* Cm01 (32.2 Mb), *M*. *robertsii* ARSEF23 (39 Mb), and *M*. *acridum* CQMa102 (38.1 Mb) (Fig. 2A). However, JEF-007 had a scaffold number of 39, whereas *Bb* ARSEF2860 had a relatively higher number (242 scaffolds). The scaffold numbers of the two *Metarhizium* species were 176 and 241. There were 10,857 protein-coding genes in JEF-007, which is quite similar but slightly more than in *Bb* ARSEF2860 (10,366), *C*. *militaris* Cm01 (9,684), *M*. *robertsii* ARSEF23 (10,582), and *M*. *acridum* CQMa102 (9,849). The KEGG pathway analysis of the Bb JEF-007 genes showed that the major pathways were metabolism (287 genes), biosynthesis of secondary metabolites (118), biosynthesis of antibiotics (86), microbial metabolism in diverse environments (69), and biosynthesis of amino acids (45) (Supplementary Fig. S2). *Bb* JEF-007 shared 6,035 ortholog genes with the other analyzed entomopathogenic fungi, but 33 ortholog genes were only found in *Bb* JEF-007 (Fig. 2B). When the *Bb* JEF-007 genes were categorized based on species, 9,346 genes (86.1%) were annotated to *B*. *bassiana*, and 804 genes (7.4%) were annotated to *Cordyceps* spp. (Supplementary Table S2). A small number of *Bb* JEF-007 genes were annotated to *Metarhizium* (47 genes, 0.4%), *Isaria* (70 genes, 0.6%), and *Verticillium* (3 genes, 0.03%).

### Comparative genetic analysis of *Bb* JEF-007 with ARSEF2860

**Figure 3:**
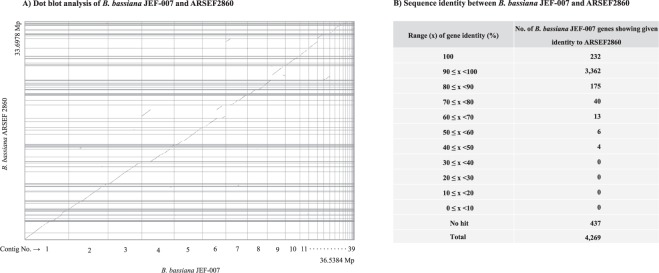
Dot plot analysis of *B*. *bassiana* JEF-007 and ARSEF2860 to investigate their syntenic relationship (**A**) and percentage of sequence identity between the genes of *B*. *bassiana* JEF-007 and ARSEF2860 (**B**).

From a syntenic relationship standpoint, the contigs of *Bb* JEF-007 were quite similar to those of *Bb* ARSEF2860 (Fig. 3A). However, some regions of *Bb* JEF-007 contig 2, 4, 6, 7, 9, and 14 showed little syntenic identity with *Bb* ARSEF2860. These un-matched *Bb* JEF-007 sequences could be an evidence that JEF-007 has larger genome than ARSEF2860. From the self-alignment of *Bb* JEF-007 using Mummer program, there were some duplications, but interestingly it was confirmed that repeat sequences (which existed as a part of one contig) formed a new contigs (Supplementary Fig. S3). When *Bb* JEF-007 genes were compared with *Bb* ARSEF2860, a total of 232 genes showed 100% identity to ARSEF2860, 3,362 genes showed 90~100% identity to ARSEF2860, and 175 genes showed 80~90% identity (Fig. 3B). A total of 63 genes in JEF-007 showed less than 80% identity to the ARSEF2860 isolate.

### Comparative biological characterization of *Bb* JEF-007 and ARSEF2860

**Figure 4:**
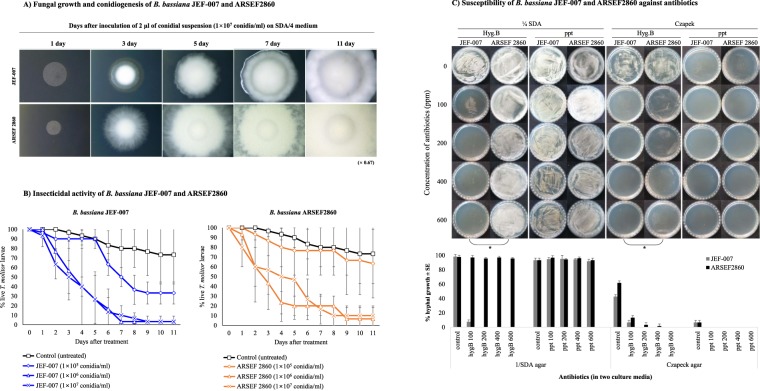
Fungal growth and conidiogenesis of *B*. *bassiana* JEF-007 and ARSEF2860 on SDA/4 medium (**A**), insecticidal activity of *B*. *bassiana* JEF-007 and ARSEF2860 against *T*. *molitor* larvae under laboratory conditions (**B**), and susceptibility of *B*. *bassiana* JEF-007 and ARSEF2860 against hygromycin B (HygB) and phosphinothricin (ppt) at 100 ~ 600 ppm on SDA/4 and Czpeck agar (**C**) in 7 days after inoculation. Fungal growth was quantitatively scored given the growth of the non-treated control and statistically analyzed (ANOVA) and represented a significant different growth with an asterisk (*).

When *Bb* JEF-007 was compared with *Bb* ARSEF2860 in morphology, ARSEF2860 showed faster hyphal growth and conidial production (Fig. 4A). However, when the same amounts of conidia (10^5^, 10^6^, and 10^7^ conidia/ml) were sprayed on the *T*. *molitor* larvae under laboratory conditions, overall *Bb* JEF-007 showed a higher virulence against the larvae compared to ARSEF2860 (Fig. 4B). Lastly, the two isolates showed different susceptibility to antibiotics, such as HygB and phosphinothricin (ppt) (Fig. 4C). In the SDA/4 agar medium with HygB, JEF-007 was more susceptible than ARSEF2860 (*F*_9,20_ = 2148.2, *p* < 0.001), and this susceptibility significantly increased when the two isolates were cultured on Czapek agar with HygB (*F*_9,20_ = 290.7, *p* < 0.001). However, for ppt, the two isolates did not show any significant susceptibility on the SDA/4 agar medium (*F*_9,20_ = 0.7, *p* = 0.622); when they were cultured on Czapek agar with ppt, they both showed no hyphal growth.

### Expression levels of high identity genes

**Figure 5:**
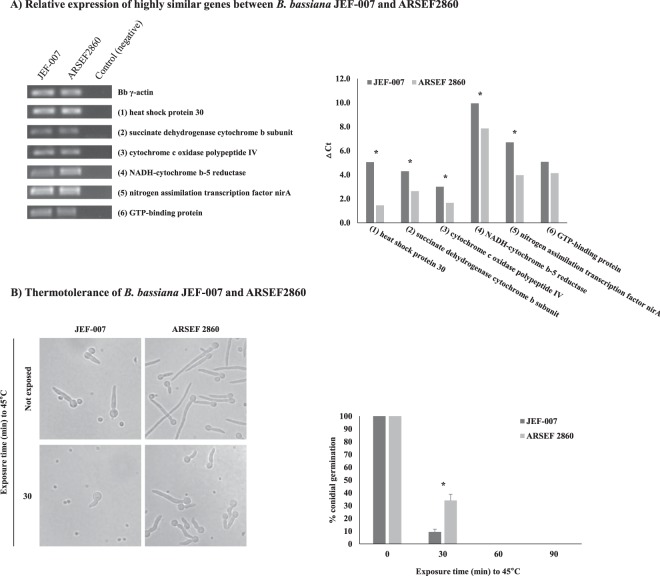
Relative expression of five highly similar genes between *B*. *bassiana* JEF-007 and ARSEF2860 (**A**) and thermotolerance of JEF-007 and ARSEF2860 when exposed to 45 °C for 30, 60, and 90 min (**B**). Asterisk (*) represents a significant difference of data between the two *Bb* isolates after t-test (p < 0.05). Full-length gels of qRT-PCR products (**A**) are presented in Supplementary Fig. S5.

To analyze the expression of genes in JEF-007 that showed a high identity (ca. 90~100%) to ARSEF2860 (Supplementary Table S3), first a reverse transcription (RT) PCR was conducted with six genes (heat shock protein 30, succinate dehydrogenase cytochrome b subunit, cytochrome c oxidase polypeptide IV, NADH-cytochrome b-5 reductase, nitrogen assimilation transcription factor nirA, and GTP-binding protein) and followed by qRT PCR. Transcription of the six genes were confirmed in the RT-PCR (Supplementary Fig. S4). Mostly ARSEF2860 genes were highly expressed compared to JEF-007 genes (Fig. 5A). Of the six analyzed genes, heat shock protein 30 (*hsp30*) of ARSEF2860 showed a higher transcription level than *hsp30* of JEF-007. A thermotolerance assay was conducted to validate the higher expression of the ARSEF2860 *hsp30* gene (Fig. 5B). After 30 minutes of exposure to 45 °C, germination was 34% in ARSEF2860, whereas it was only 9% in JEF-007. As presented in the figure, hyphal growth was faster in ARSEF2860 under normal conditions without heat exposure.

### Sequence variations of five pathogenesis-related genes

**Figure 6:**
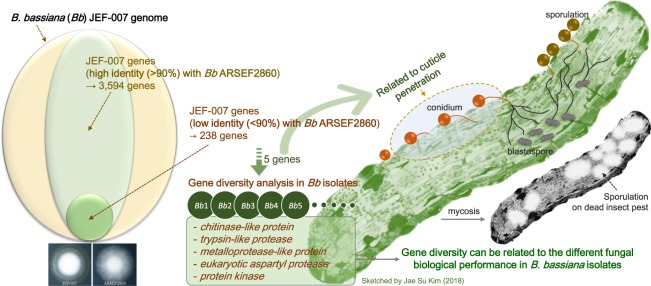
Schematic diagram to explain the possible relationship of the selected five genes from the low identity *B*. *bassiana* JEF-007 gene group with fungal pathogenesis and importance of the selected gene-based gene diversity analysis in several *B*. *bassiana* isolates.

**Figure 7:**
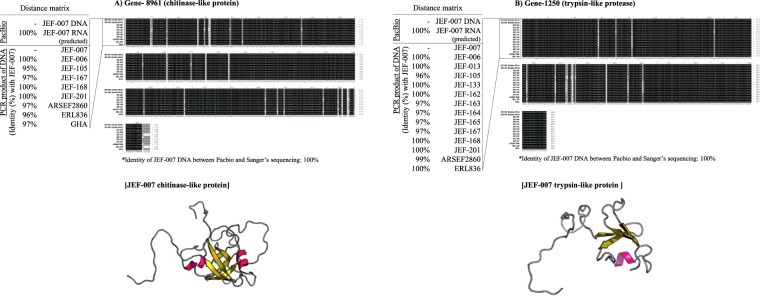
Highly conserved *chitinase-like* and *trypsin-like protease* genes among *B*. *bassiana* JEF-007 and other *Bb* isolates. (**A**) *chitinase-like protein* gene and (**B**) *trypsin-like protease* gene. PacBio sequencing DNA and predicted RNA data were aligned to investigate similarity and the existence of introns. Sanger sequences of PCR DNA were aligned among the *B*. *bassiana* isolates, and similarity results are provided. 3D protein structures of JEF-007 were generated using RaptorX.

**Figure 8:**
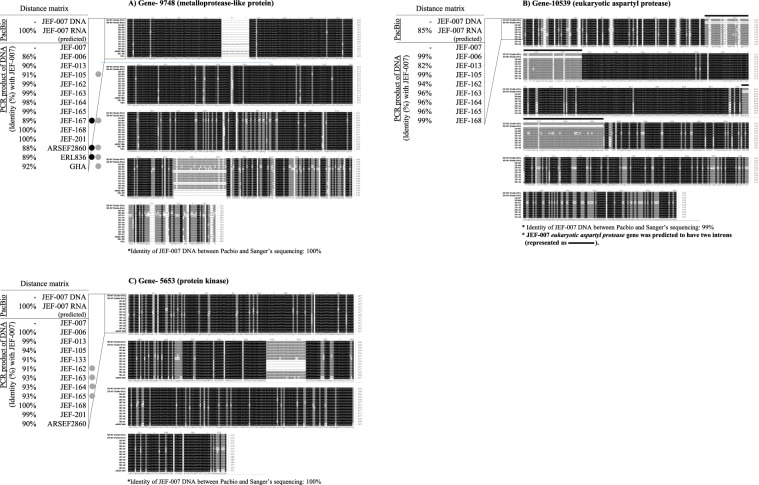
Diverse sequences of metalloprotease-like protein, eukaryotic aspartyl protease, and protein kinase genes in B. bassiana JEF-007 and other Bb isolates. (**A**) metalloprotease-like protein gene, (**B**) eukaryotic aspartyl protease gene, and (**C**) protein kinase gene. PacBio sequencing DNA and predicted RNA data were aligned to investigate similarity and the existence of introns. Sanger sequences of PCR DNA were aligned among the B. bassiana isolates, and similarity results are provided. Circles represent B. bassiana isolates including insertion (●) and deletion () fragments, compared to JEF-007. JEF-007 eukaryotic aspartyl protease gene was predicted to have two introns.

When *Bb* JEF-007 genes which were annotated as *Bb* ARSEF2860 were divided to high identity and low identity groups, some of low identity genes showing possible relation to cuticle penetration were subjected to a gene diversity analysis using ca. 10 *B*. *bassiana* isolates (Fig. 6). The five low identity genes (Supplementary Table S3) could be grouped into two categories. DNA sequence was highly conserved in the group including *chitinase-like* and *trypsin-like protease* genes (Fig. 7). The other group of genes, which included *metalloprotease-like protein*, *eukaryotic aspartyl protease*, and *protein kinase*, showed a diversity of sequence when compared to the highly conserved group (Fig. 8). The only gene thought to contain introns out of those selected was *eukaryotic aspartyl protease* gene.

#### Chitinase-like protein (gene code: Bb-00008961)

The Sanger DNA sequencing of the JEF-007 *chitinase-like protein* was identical to the PacBio sequencing data. When the JEF-007 *chitinase-like protein* sequence was compared to eight other *B*. *bassiana* isolates (JEF-006, JEF-105, JEF-167, JEF-168, JEF-201, ARSEF2860, ERL836, and GHA), the similarity was high, 96~100%, in all those analyzed (Fig. 7A).

#### Trypsin-like protease (gene code: Bb-00001250)

The Sanger DNA sequencing of the JEF-007 *trypsin-like protein* was identical to the PacBio sequencing data. The JEF-007 *trypsin-like protein* sequence had a high similarity, 96~100%, to 13 other *B*. *bassiana* isolates (JEF-006, JEF-013, JEF-105, JEF-133, JEF-162, JEF-163, JEF-164, JEF-165, JEF-167, JEF-168, JEF-201, ARSEF2860, and ERL836) (Fig. 7B).

#### Metalloprotease-like protein (gene code: Bb-00009748)

The Sanger DNA sequencing of the JEF-007 *metalloprotease-like protein* was identical to the PacBio sequencing data. When the JEF-007 metalloprotease-like protein sequence was compared to 12 other *B*. *bassiana* isolates, a relatively high similarity of more than 95% was found in the analyzed isolates except JEF-006, JEF-013, JEF-105, JEF-167, ARSEF2860, ERL836, and GHA (Fig. 8A). In particular, JEF-167, ARSEF2860, and ERL836 showed insertional DNA sequences, about 21 bp long, and these sequences were identical among the three isolates. When compared to JEF-007, deleted sequences were observed in the *metalloprotease-like protein* sequence of JEF-105, JEF-167, ARSEF2860, ERL836, and GHA.

#### Eukaryotic aspartyl protease (gene code: Bb-00010539)

Based on the JEF-007 PacBio sequencing, the gDNA of *eukaryotic aspartyl protease* was similar to the mRNA sequence; however, two distinct introns were found in the DNA structure (Fig. 8B). The first intron was 69 bp long, and the second intron was 64 bp long. The Sanger DNA sequencing of the JEF-007 *eukaryotic aspartyl protease* was 99% identical to the PacBio sequencing data, but a 130 bp sequence of the 5′ fragment could not be read. When the JEF-007 eukaryotic aspartyl protease sequence was compared in eight other *B*. *bassiana* isolates, sequences were trimmed based on the similarity level. A relatively high similarity of more than 95% was found in the eight analyzed isolates except JEF-013 (82% identity with JEF-007).

#### Protein kinase (gene code: Bb-00005653)

The Sanger DNA sequencing of the JEF-007 *protein kinase* was identical to the PacBio sequencing data. When the JEF-007 sequence was compared to 11 other *B*. *bassiana* isolates, a high similarity of 99~100% was shown by only four isolates (JEF-006, JEF-013, JEF-168, and JEF-201). However, seven isolates (JEF-105, JEF-133, JEF-162, JEF-163, JEF-164, JEF-165, and ARSEF2860) showed a relatively lower similarity of about 91~94% (Fig. 8C).

## Discussion

A species of *B*. *bassiana* has received interest, and several isolates have been deeply studied in an application to control pests in agriculture and forests. Additional work, such as determining the mode of action and virulence-related genetic factors, is being conducted and some outcomes have been reported^37,38^. At present, 10 *B*. *bassiana* isolates have been fully sequenced, and their genomes are available in GenBank (https://www.ncbi.nlm.nih.gov/genome). The 10 isolates including *Bb* JEF-007 are ARSEF2860 (33. 7 Mb, 2004), ARSEF1520 (37.0 Mb, 2016), ARSEF2597 (38.8 Mb, 2016), ARSEF5078 (34.4 Mb, 2016), ARSEF4305 (34. 8 Mb, 2016), D1–5 (36.7 Mb, 2014), ARSEF8028 (35.0 Mb, 2016), BCC2660 (34.6 Mb, 2017), JEF-007 (36.5 Mb, 2018), and IMV00265 (35.2 Mb, 2017). Most of the whole genome sequencing has been completed from 2014~2018 owing to the development of new generation sequencing (NGS) technology. The scaffold numbers possibly depend on the sequencing technique, but the lowest number was 12 in AERSEF8028, and the highest was 3,365. In this work, there were 35 scaffolds in JEF-007, which was relatively fewer than in the others. The PacBio sequencing may have had some errors in sequencing due to the long length reads, but could possibly predict a more complete whole genome^39^. Given the general size of a fungal chromosome (>1 Mb)^40^, JEF-007 may have about 10 chromosomes, but this needs to be further clarified. In our effort, providing the exact number of chromosome was not easy because a physical map should be built via karyotyping. Karyotyping is popular in human and animal cells to figure out the features of chromosomes, but not in fungal cells. Alternatively, a pulsed field gel electrophoresis could be used to investigate the fungal chromosome numbers, but not feasible all the times. Herein this manuscript, given the objective of this work, we just tried to predict the chromosome number of JEF-007, rather than providing the exact chromosome number. From the KEGG pathway analysis of Bb JEF-007, most major pathways are possibly involved in the cell growth of the next generations and infection of the target insects when this fungus encounters a favorable environment.

In this summary of genome features, *Bb* JEF-007 has a 36.5 Mb genome size, and 10,857 genes were predicted, which is similar to previously sequenced entomopathogenic fungi, such as *C*. *militaris*, *M*. *rosertsii*, and *M*. *acridum*^41,42^. The average genome size of Ascomycota species is about 36 Mb^40^. In this work, the five represented entomopathogenic fungi, including *Bb* JEF-007, may possibly share many ortholog genes based on the reciprocal analysis. The two *B*. *bassiana* isolates JEF-007 and ARSEF2860 share many ortholog genes, but about 174 genes were observed only in JEF-007, which may be important in describing the unique characteristics of JEF-007 compared to ARSEF2860, such as virulence against insects and growth-related cell cycling. In particular, 33 ortholog genes were unique when compared to the other four entomopathogenic fungi, and this group of genes could be used as feasible markers to identify *Bb* JEF-007. However, if the *Bb* JEF-007 genome is compared to other *B*. *bassiana* isolates recently registered in GenBank as described above or un-released *B*. *bassiana* genomes, the number of unique genes could be changed. When the predicted genes of *Bb* JEF-007 were annotated to the GenBank database, about 86% were annotated to *B*. *bassiana*, and 7.5% of the genes (ca. 804 genes) were annotated to *Cordyceps* species, which suggests a strong genetic relationship between *Bb* JEF-007 and *Cordyceps*. A recent report suggests that the sexual life cycle of *B*. *bassiana* is similar to that of *Cordyceps*, particularly in *C*. *bassiana* although it is rarely observed^43^. Interestingly, 0.6% of the genes (ca. 70 genes) were related to *Isaria* species.

Our main focus was to investigate how much the *Bb* JEF-007 genome differed from that of *Bb* ARSEF2860. In the dot blot analysis to determine the syntenic relationship between *Bb* JEF-007 and *Bb* ARSEF2860, most JEF-007 gDNA were placed at the same location (probably in the same chromosome) in ARSEF2860, showing very high synteny, except in less than 10% of the genomic DNA, which might provide a hint in predicting the evolutionary event in *B*. *bassiana* species^44^. In this work, a clear identification of chromosomes was not conducted; however, small numbers of JEF-007 genes could be localized in different chromosomes of ARSEF2860, but this needs to be clarified. When the genes of *Bb* JEF-007 were subjected to BlastX, a large number of genes (78% of the total = 3,594 genes) showed more than 90% identity with *Bb* ARSEF2860 genes. Interestingly, the expression level of highly identical genes could be different between the two *Bb* isolates^45^, which was proved in this work with a *heat shock protein* gene. However, as described above, our main focus was given to the genes that showed a low identity less than 80% (about 238 genes) in the two *Bb* isolates. Genes that received no hits were not considered in this work because they might have occurred from sequencing errors or inappropriate annotation. In the sequence identity analysis, JEF-007 sequences which were annotated as the description of *B*. *bassiana* ARSEF2860 (including the word of “ARSEF2860” in the annotation) were selected and summarized. In this screening process, many JEF-007 genes which were annotated as *B*. *bassiana* D1–5 or other *B*. *bassiana* isolates were removed although they might be shared with ARSEF2860. One of the reasons of this screening was to minimize the number of shared *Bb* JEF-007 genes with other *B*. *bassiana* isolates and select high and low similarity genes between the two isolates (*Bb* JEF-007 and ARSEF2860).

The genomic difference between *Bb* JEF-007 and *Bb* ARSEF2860 could result in different morphological growth phenotypes, and biological performances^46^. As described above in the result section, two isolates had a different growth rate and different speed of conidiogenesis, which might be a consequence of genetic differences. The group of genes with a low identity could be used to describe this morphological difference. Secondly, ARSEF2860 showed higher virulence against the *T*. *molitor* larvae, compared to *Bb* JEF-007. In pathogenesis, conidial germination- and hyphal penetration-related genes are possibly very similar to each other, but the difference in virulence in this study could be explained by the different expression levels. Alternatively, some of the key pathogenesis-related genes^14,47^ could be diverse in sequences, possibly causing different levels of virulence. Lastly, the two isolates JEF-007 and ARSEF2860 showed different susceptibility to chemicals, such as hygromycin B (HygB) and phosphinothricin (ppt). In a previous work, *B*. *bassiana* isolates showed variations in susceptibility to HygB^35^. Overall, ARSEF2860 showed stronger resistance to the two antibiotics. Interestingly, nutritional support increased resistance to the chemicals. This susceptibility could be explained by the genetic difference or different expression level of metabolism-related genes^48^. Additionally, specific genes in each *B*. *bassiana* JEF-007 and ARSEF2860 were analyzed. In more details, the amino acid sequences of the two isolates were aligned and un-matched sequences were listed up and finally blast-x of un-matched sequences was conducted using nr database for identifications. The two *B*. *bassiana* genomes have been already registered in the database, so the un-matched genes showed ca. 100% identity and specific genes in each isolate were almost annotated as hypothetical proteins **(**Supplementary Shreadsheet: Dataset 1**)**. A further analysis needs to verify the functions of the specific genes.

Six randomly selected highly similar genes were subjected to qRT-PCR to determine the expression levels; overall, the gene expression levels of ARSEF2860 were relatively higher than JEF-007. Although all the six genes could be more deeply investigated, we focused on *heat shock protein 30*, which might be related to fungal thermotolerance^49,50^. In fact, a higher thermotolerance was found in ARSEF2760 compared to JEF-007. The *heat shock protein 30* gene was 100% identical to that in ARSEF2860; therefore, it was assumed that their expression levels could be different. Transcription factors or promoters for the *heat shock protein* gene could be deeply studied in future research. Real-time PCR indicated that the expression of other genes, including succinate dehydrogenase, cytochrome oxidase, NADH-cytochrome reductase, nitrogen assimilation transcription factor, and GTP-binding proteins, which are known to be important for cell metabolism, such as the TCA and electron transport system, were much higher in ARSEF2860, which might be related to faster fungal growth and conidiogenesis^51,52^.

Five JEF-007 genes with a low identity in ARSEF2860 were aligned to other *B*. *bassiana* isolates (ca. 10 isolates) to investigate their sequence diversity within the same species. Interestingly, the *chitinase-like protein* sequence (gene 8961) and *trypsin-like protease* sequence (gene 1250) of JEF-007 were highly similar (conserved) to the other analyzed *B*. *bassiana* isolates. These could be regarded as key enzymes to penetrate insect cuticles throughout most of *B*. *bassiana* isolates, as studied in other fungal species^53^. However, their expression level, which is possibly related to fungal virulence, might be diverse depending on the isolates.

Three other genes, *metalloprotease-like protein*, *eukaryotic aspartyl protease*, and *protein kinase*, showed relatively high sequence variations. The *metalloprotease-like protein* gene sequence (gene 9748) of JEF-007 was definitely different from that of six *B*. *bassiana* isolates including ARSEF2860. Additions or deletions of a short fragment were observed in the *metalloprotease-like protein* gene of the six isolates, which might be related to a diverse phenotype and biological functions. In regard to *eukaryotic aspartyl protease* gene sequence (gene 10539) of JEF-007, two other *B*. *bassiana* isolates (JEF-013 and JEF-162) showed a difference in sequence from JEF-007. The virulence of the two isolates could be decreased or increased when compared to JEF-007. It seems likely that there were two introns in the gene of the *eukaryotic aspartyl protease* in JEF-007, but the two possible introns and alternative splicing need to be clarified in other *B*. *bassiana* isolates. There were some unsequenced fragments in upstream of the *eukaryotic aspartyl protease*, which might have resulted from inappropriate primer design or a misreading problem in the PacBio sequencing, which is an issue to be resolved in a following study. Lastly, *protein kinase* (gene 5653) sequences were significantly different in seven of the *B*. *bassiana* isolates; in particular, a deletion of a short fragment was found in JEF-162, JEF-163, JEF-164, and JEF-165. This sequence diversity might be related to the different levels of phosphorylation of enzymes in pathogenesis and cell cycling.

Our assumption based on the five genes might not cover the overall features of the fungal biological performance, but the five genes are reported as important pathogenesis-related genes and so our analyses could provide a milestone of a further deep understanding. From the examination of sequence diversity in *B*. *bassiana*, if the variation between *B*. *bassiana* strains might be quite high, the definition of a species and the evolutionary process need to be seriously reconsidered. The final figures showing sequence diversity possibly illustrate how the genome sequence data could be used to set up a taxonomic system of intra- or interspecies for describing fungal diversity in entomopathogenic fungi.

In conclusion, the whole genome of *B*. *bassiana* JEF-007 was sequenced and analyzed to reveal that *Bb* JEF-007 has a genome size of 36.5 Mb with 10,857 protein-coding genes. These genomic features of JEF-007 were quite similar to those of other well-known entomopathogenic fungi, such as *Metarhizium* and *Cordyceps*. When the genetic difference between *Bb* JEF-007 and *Bb* ARSEF2860 were compared, many genes were identical between the two *Bb* isolates, but their expression levels could be different and possibly relate to biological performance. One more important finding in this work was that some genes of JEF-007, such as *chitinase* and *trypsin-like protease*, involved in pathogenesis, were highly conserved, while other genes showed noticeable sequence diversity within the same species. Given the genetic diversity of genes in *B*. *bassiana* available for biological control of agricultural pests, selection of highly virulent isolates with thermal stability and high conidial productivity is a pre-requisite, and this genetic approach could support the development of excellent isolates for industrialization.

## Methods

### Fungal strain and culture

*B*. *bassiana* (*Bb*) isolates including JEF-007 and ARSEF2860 from the Jeonbuk (Chonbuk) National University Collection of Entomopathogenic Fungi (JEF), Korea, and USDA-ARS Collection of Entomopathogenic Fungal Cultures (ARSEF) were cultured and maintained on one-quarter strength Sabouraud dextrose agar (SDA/4; Difco, Lawrence, KS, USA) in darkness at 25 °C for colony growth^54^. To produce fungal spores (conidia), the *B*. *bassiana* isolates were inoculated on SDA/4 at 100 μl (1 × 10^7^ conidia ml^−1^) per 60-mm diameter. After incubation in darkness at 25 °C for 10 days, conidia (with hyphae removed) were harvested, suspended in 0.03% (v/v) siloxane solution (Silwet L-77; FarmHannong, Seoul, Korea) as a wetting agent, and adjusted to 1 × 10^7^ conidia ml^−1^.

### Pathogenesis and virulence assay against insect pests

*Bb* JEF-007 was assayed against six agricultural insect pests, western flower thrips, citrus flatid planthopper, fall webworm, bean bug, mealworm and persimmon fruit moth from laboratory colonies or field-collected populations to investigate its pathogenicity. Adults, nymph or larval stages of the insects were placed in a 90-mm Petri dish with moisturized filter paper (100 μl distilled water). The fungal conidial suspension (1 × 10^7^ conidia/ml) was sprayed on the stages at 2 ml/dish, and the dishes were kept at 25 ± 1 °C (60% RH) for 7~10 days. Secondly *Bb* JEF-007 and *Bb* ARSEF2860 were bio-assayed against *T*. *molitor* (mealworm) larvae to compare their virulence. A colony of *T*. *molitor* was obtained from the National Institute of Agricultural Sciences, Korea. The *T*. *molitor* larvae were kept in screened cages at 25 ± 1 °C with 60% relative humidity (RH) and a 16 h:8 h (L:D) photoperiod, with Chinese cabbage and wheat bran provided as food. Ten (10) third instar larvae were placed in a 90-mm Petri dish with moisturized filter paper with three replicates. The fungal conidial suspensions (1 × 10^5^, 1 × 10^6^ and 1 × 10^7^ conidia/ml) were sprayed on the larvae at 2 ml/dish. The larvae were kept at 25 ± 1 °C (60% RH) after they were sprayed. The surviving *T*. *molitor* larvae were daily counted for 11 days.

### Fungal thermotolerance

For the thermotolerance assay, the fungal isolates were cultured on SDA/4 medium at 25 ± 1 °C in darkness for 7 days. A mycotized agar disk (6 mm diameter) was aseptically collected from each culture using a sterile cork-borer and placed separately in an Eppendorf tube containing 1 ml of 0.03% (v/v) siloxane solution (Silwet L-77, FarmHannong, Korea)^24^. The tube was vortexed for 1 min and then placed in an incubator at 45 °C for 0 (before exposure), 30, 60, 90, and 120 min. Each heat-exposed fungal isolate was cultured on SDA/4 medium and incubated at 25 ± 1 °C for 18 h. The percentage of germinated isolates was determined by randomly counting the number of germinated and un-germinated conidia among 100 counts under a microscope (400×). Each treatment was replicated three times.

### Antibiotics susceptibility

The susceptibility of *B*. *bassiana* isolates JEF-007 and ARSEF2860 to hygromycin B (HygB) and phosphinothricin (ppt) was investigated on agar plates as in a previous report^36^. Conidial suspensions (1 × 10^7^ conidia ml^−1^) of the isolates were spread on SDA/4 or Czapek’s agar medium containing 100, 200, 400 and 600 μg ml^−1^ HygB or ppt with three replicates. Fungal growth was observed at 3, 5, and 7 days after inoculation and it was quantitatively scored given the fungal growth of the non-treated control and statistically analyzed (ANOVA).

### PacBio RS for whole genome sequencing

Genomic DNA of *B*. *bassiana* JEF-007 was extracted from 7-day-old mycelial masses using a DNA extraction kit (MG^TM^ gDNA purification kit (protocol for Yeast), www.macrogen.com). *B*. *bassiana* 18S rDNA fragment served as an internal control (*Bb* 18S-F: 5′-TTA CGT CCC TGC CCT TTG TA-3′/*Bb* 18S-R: 5′-CCA ACG GAG ACC TTG TTA CG-3′). PCR amplifications were performed as follows on a C-1000 Thermal Cycler (Bio-Rad, Hercules, CA, USA): preheating at 95 °C for 5 min followed by 30 cycles of 94 °C for 30 s, 57 °C for 30 s, and 72 °C for 30 s, followed by a 10 min final extension at 72 °C and storage at 12 °C. PCR products were analyzed by electrophoresis on 0.8% agarose gels in 1 × TBE buffer. For PacBio RS sequencing in Macrogen (www.macrogen.com), 8 g of input gDNA was used for 20 kb library preparation. For gDNA shorter than 17 kb, a Bioanalyzer 2100 (Agilent, Santa Clara, CA, USA) was used to determine the actual size distribution. The gDNA was sheared with g-TUBE (Covaris Inc., Woburn, MA, USA) and purified using AMPure PB magnetic beads (Beckman Coulter Inc., Brea, CA, USA) if the apparent size was greater than 40 kb. The gDNA concentration was measured using both a NanoDrop spectrophotometer and a Qubit fluorometer, and approximately 200 ng/μL of gDNA was run on a field-inversion gel. A total 10 μl library was prepared using a PacBio DNA Template Prep Kit 1.0 (for 3~10 kb). SMRTbell templates were annealed using PacBio DNA/Polymerase Binding Kit P6. The PacBio DNA Sequencing Kit 4.0 and 8 SMRT cells were used for sequencing. SMRT cells (Pacific Biosciences) using C4 chemistry and 240 min movies were captured for each SMRT cell using the PacBio RS II (Pacific Biosciences, Menlo Park, CA, USA) sequencing platform by Macrogen (Seoul, Korea). The subsequent steps are based on the PacBio Sample Net-Shared Protocol, which is available at http://pacificbiosciences.com.

### *De novo* assembly

The subreads were assembled using FALCON (v0.2.1) to obtain assembled DNA sequence data. The FALCON program is utilized for *de novo* assembly based on the PacBio platform data. The assembled DNA sequence was corrected by Quiver (v1), and an error correction step was performed through SMRTpipe (v2.3.0.139497). To obtain genome-guided transcriptome assembly data, RNA reads were mapped to assembled DNA sequence using Tophat (v2.0.13)^55^, and an assembled transcriptome sequence was obtained from the resulting BAM file using Trinity (r20140717)^56^. Assembled DNA sequence and assembled transcriptome sequence data were annotated using a Seqping (v0.1.33) pipeline^57^. This pipeline builds a gene prediction model (GlimmerHMM (v3.0.4)^58^, AUGUSTUS (v3.2.2)^59^, and SNAP (2012/05/17)). Prediction results are then combined with the annotation program MAKER (v2.28)^60^ contained in the Seqping pipeline. For additional annotation, the consensus sequences were searched against the GenBank non-redundant (NR) database using blastx (v2.4.0+)^61^. An analysis was conducted to confirm pathways involved in the protein-coding gene sequences obtained as a result of the annotation, an analysis was performed using the KEGG database (http://www.genome.jp/tools/kaas/)^62^.

### Comparison of genome features between JEF-007 and others

For comparisons of *Bb* JEF-007 with the other four entomopathogenic fungi, *B*. *bassiana* ARSEF2860, *Cordyceps militaris* (*Cm*) Cm01, *Metarhizium robertsii* (*Mr*) ARSEF23, and *Metarhizium acridum* (*Ma*) CQMa102^41,63^, various numerical values were obtained using assembly statistics and tools. Repeat masker (v4.0.5) was used to obtain repeat rate^64^, and TMHMM (v2.0) was used to obtain secreted protein proportion^65^. A self-alignment analysis was conducted using Mummer (http://mummer.sourceforge.net) to see duplication of the sequences in *B*. *bassiana* JEF-007.

### Reciprocal BLAST analysis and dot blot analysis

OrthoMCL (v2.0.3) was used to identify orthologous and paralogous gene clusters between the *Bb* JEF-007 sample and other entomopathogenic fungi^66^. In the analysis, data of the four entomopathogenic fungi *Bb* ARSEF2860, *Cm* Cm01, *Mr* ARSEF23, and *Ma* CQMa102 were used. After removing protein sequences shorter than 10 amino acids or with a percentage of stop codons greater than 20%, all-versus-all analysis was performed with blastp (v2.2.25+) (E value threshold 1e-5). The protein pairs from the blastp results were processed with the value of inflation factor 1.5 to meet the balance of sensitivity and selectivity using OrthoMCL (mcl-12-068). This result was separated into orthologous and paralogous gene clusters to be used in further analysis. Dot blot analysis figures were drawn with Assemblytics^67^ to compare *Bb* JEF-007 sample sequences with ARSEF2860 strain sequences. In the analysis, Nucmer program from the MUMer package (http://mummer.sourceforge.net) was used and specific setting was maxmatch (use all anchor matches regardless of their uniqueness), mincluster 500 and minmatch 100. In the dot plot analysis, option of unique sequence length required was 10,000.

### Transcriptional validation of high and low identity genes

From the annotation of the *Bb* JEF-007 genes to the previously reported *Bb* ARSEF2860 genes, six JEF-007 genes showing high identity to ARSEF2860, heat shock protein 30, succinate dehydrogenase cytochrome b subunit, cytochrome c oxidase polypeptide IV, NADH-cytochrome b-5 reductase, nitrogen assimilation transcription factor nirA, and GTP-binding protein were randomly selected (Supplementary Table S3) and subjected to a reverse transcription (RT) PCR and a real-time PCR using primers (Supplementary Table S4). Secondly, five JEF-007 genes showing low identity to the ARSEF2860 and probably high correlation to fungal pathogenesis were selected as follows: trypsin-like protease, metalloprotease-like protein, chitinase-like protein, eukaryotic aspartyl protease, and protein kinase genes (Fig. 6). It was not always successful to clearly read more than six genes in the low identity group using the Sanger’s method. The sequences of *B*. *bassiana* JEF-007 genes were compared to other ca. 10 *B*. *bassiana* isolates by Sanger sequencing (PCR) with primers (Supplementary Table S5) which annealed to ca. 100 bp outer area of the target protein coding sequence (analyzed by Bowtie 1.2.2 program (http://bowtie-bio.sourceforge.net), and aligned to determine DNA-based sequence diversity (GeneDoc ver. 2.6, http://genedoc.software.informer.com/2.6/). In each gene, PacBio-based JEF-007 DNA and predicted RNA sequences were aligned to check the existence of introns, and Sanger sequences of *B*. *bassiana* isolates (ca. 10) including JEF-007 were aligned to see the gene diversity, such as substitution, insertion (●) and deletion () fragments. The 3D structure of JEF-007 chitinase-like and trypsin-like proteins were generated using RaptorX server (http://raptorx.uchicago.edu).

## Electronic supplementary material

Supplementary Information

Dataset 1
