# Publisher Correction: Genomic Analysis of the Insect-Killing Fungus *Beauveria bassiana* JEF-007 as a Biopesticide

**DOI:** 10.1038/s41598-021-92483-7

**Published:** 2021-07-05

**Authors:** Se Jin Lee, Mi Rong Lee, Sihyeon Kim, Jong Cheol Kim, So Eun Park, Dongwei Li, Tae Young Shin, Yu-Shin Nai, Jae Su Kim

**Affiliations:** 1 Department of Agricultural Biology, College of Agriculture & Life Sciences, Chonbuk National University, Jeonju, 54596 Korea; 2 Department of Biotechnology and Animal Science, National Ilan University, Ilan, Taiwan; 3 Plant Medical Research Center, College of Agricultural and Life Sciences, Chonbuk National University, Jenoju, 54596 Korea

Correction to: *Scientific Reports* 10.1038/s41598-018-30856-1, published online 17 August 2018

The original version of this Article contained an error in Figure 1 where the panel for the Bb JEF-007-infected Bean bug (*Riptortus pedestris*) contained white lines. The original Figure 1 and accompanying legend appear below.

**Figure 1:**
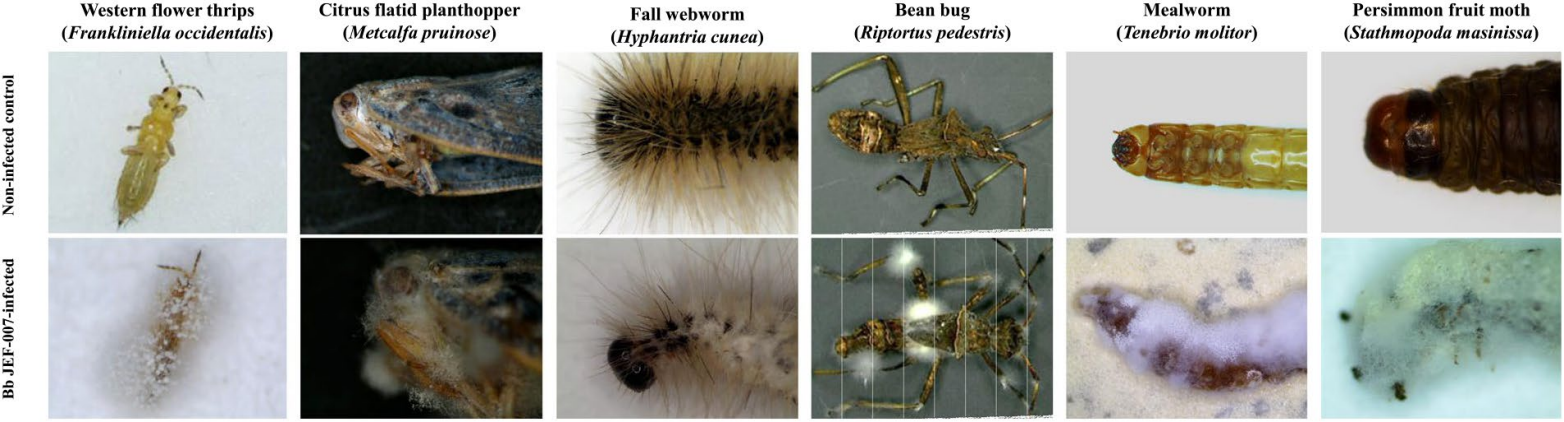
Insecticidal activity (virulence) of *B*. *bassiana* JEF-007 against western flower thrips, citrus flatid planthopper, fall webworm, bean bug, mealworm and persimmon fruit moth in laboratory conditions. The JEF-007 conidial suspension (1 × 10^7^ conidia/ml) was sprayed on the adult, nymph or larva at 2 ml/dish, and the dishes were kept at 25 ± 1 °C (60% RH) for 7 ~ 10 days. The above photos were taken by the authors of this article.

The original Article has been corrected.

