# Supplementary material for: Genomic Analysis of the Insect-Killing Fungus Beauveria bassiana JEF-007 as a Biopesticide
Source: Sci Rep. 2018 Aug 17;8:12388. doi: 10.1038/s41598-018-30856-1 (PMC6098154; doi:10.1038/s41598-018-30856-1)
Supplement: Supplementary file 1 — Supplementary Information [file 41598_2018_30856_MOESM1_ESM.pdf]

Supplementary Information

Genomic Analysis of the Insect-Killing Fungus *Beauveria bassiana* JEF-007 as a Biopesticide

Se Jin Lee[1†], Mi Rong Lee[1†], Sihyeon Kim[1], Jong Cheol Kim[1], So Eun Park[1], Dongwei Li[1], Tae Young Shin[1], Yu-Shin Nai[2] and Jae Su Kim[1,3*]

[1]Department of Agricultural Biology, College of Agriculture & Life Sciences, Chonbuk National University, Jeonju 54596, Korea
[2]Department of Biotechnology and Animal Science, National Ilan University, Ilan, Taiwan
[3]Plant Medical Research Center, College of Agricultural and Life Sciences, Chonbuk National University, Jenoju 54596, Korea

[†]These two authors contributed equally to this work

Running title: Genomic analysis of *B. bassiana* JEF-007 as biopesticide

*Corresponding author: **Jae Su Kim.** Department of Agricultural Biology, College of Agriculture & Life Sciences, Chonbuk National University, Korea. Tel: +82 63 270 2525; Fax: +82 63 270 2531; and e-mail: jskim10@jbnu.ac.kr

Supplementary Table S1. *De novo* assembly of *B. bassiana* JEF-007 reads for contig prediction

No. contigs	Total Length (bp)	N_{50} (bp)	Max Length (bp)	Min Length (bp)	Average Length (bp)
39	36,538,394	3,115,233	4,836,080	5,751	936,881

Contig number	Length (bp)	GC %	Depth
Contig 1	4,836,080	47.80	104
Contig 2	4,313,377	47.20	104
Contig 3	3,721,617	48.50	104
Contig 4	3,539,716	50.20	107
Contig 5	3,115,233	46.70	102
Contig 6	2,580,292	48.60	105
Contig 7	2,356,961	50.40	107
Contig 8	2,191,280	43.90	102
Contig 9	1,987,760	50.80	107
Contig 10	1,591,247	48.70	107
Contig 11	977,447	51.20	109
Contig 12	957,547	52.20	107
Contig 13	681,700	48.10	106
Contig 14	677,945	49.60	106
Contig 15	561,348	45.90	105
Contig 16	508,980	47.0	101
Contig 17	390,418	50.30	109
Contig 18	260,236	26.70	87
Contig 19	246,642	44.20	91
Contig 20	225,477	46.40	107
Contig 21	178,896	42.30	97
Contig 22	105,676	44.80	100
Contig 23	100,976	53.40	114
Contig 24	72,605	24.50	71
Contig 25	57,124	53.70	108
Contig 26	38,876	49.70	170
Contig 27	38,659	52.30	965
Contig 28	31,210	27.0	699
Contig 29	25,046	39.70	28
Contig 30	22,032	25.70	34
Contig 31	21,250	32.40	1
Contig 32	21,017	51.60	35
Contig 33	20,327	35.50	3
Contig 34	18,324	19.0	13
Contig 35	17,304	51.30	19
Contig 36	17,020	38.40	35
Contig 37	16,589	20.70	15
Contig 38	8,409	20.30	1
Contig 39	5,751	21.0	1
Total	36,538,394	48.04	105

Supplementary Table S2. Annotation of JEF-007 genes (No: 10,847 genes) to other entomopathogenic fungal species via BLASTX in the nr database

Genus	Species	Number of *B. bassiana* JEF-007 gene
Beauveria		**9,346**
	Beauveria bassiana	9,346
Metarhizium		**47**
	Metarhizium majus	3
	Metarhizium acridum	7
	Metarhizium album	2
	Metarhizium anisopliae	15
	Metarhizium brunneum	3
	Metarhizium guizhouense	8
	Metarhizium rileyi	9
Cordyceps		**804**
	Cordyceps brongniartii	671
	Cordyceps confragosa	82
	Cordyceps militaris	51
Isaria		**70**
	Isaria farinosa	1
	Isaria fumosorosea	69
Verticillium		**3**
	Verticillium alfalfae	1
	Verticillium dahliae	1
	Verticillium longisporum	1
Fusarium	sp.	**23**
Penicillium	sp.	**11**
Other genus	-	163
No hit	-	437
**Total**	-	**10,857**

Supplementary Table S3. List of randomly selected *B. bassiana* JEF-007 genes showing different identity to *B. bassiana* ARSEF2860 genes

JEF-007 Gene (coded)	Length (bp)	Description of gene	% identity with ARSEF2860 (BlastX)
00003787	197	heat shock protein 30	100
00008353	163	succinate dehydrogenase cytochrome b subunit	100
00009843	203	cytochrome c oxidase polypeptide IV	100
00006175	303	NADH-cytochrome b-5 reductase	93
00006769	194	nitrogen assimilation transcription factor nirA	92
00005653	186	protein kinase	84
00000010	181	GTP-binding protein	83
00001250	105	trypsin-like protease	83
00009748	233	metalloprotease-like protein	80
00008961	175	chitinase-like protein	75
00010539	391	eukaryotic aspartyl protease	73

Supplementary Table S4. Primers for reverse transcription (RT) PCR and qRT-PCR of high identity genes between *B. bassiana* JEF-007 and ARSEF2860

Primers	Sequence (5'→3')	Amplicon size (bp)	Target gene
Bb 18S-F	TTA CGT CCC TGC CCT TTG TA	167	*B. bassiana* 18S rRNA
-R	CCA ACG GAG ACC TTG TTA CG		
Bb γ-actin-F	GTCAAGTCATCACCATTGGC	150	*B. bassiana* gamma actin
-R	CGTAGAGATCCTTGCGAACA		
Gene_3787-F	GAAGATGTTTCGGACAAGCC	227	Heat shock protein 30
R	GGGACCGAAACCGAGAGTAT		
Gene_8353-F	CTACTACGCAAGCTCGTCCC	252	Succinate dehydrogenase cytochrome b subunit
R	CTAGGCTCTGGGACTCAACG		
Gene_9843-F	AGTCGACTGGTCTTGAGCGT	318	Cytochrome c oxidase polypeptide IV
R	AAGTCTGAGGCTCCGGGTAT		
Gene_6175-F	GCCACGCGAGAAGAACTTAC	338	NADH-cytochrome b-5 reductase
R	ACTCTTGCCCCAATATGTCG		
Gene_6769-F	GTGCTATGCGCAGCTTTACA	314	Nitrogen assimilation transcription factor nir A
R	CGAGCCTGTCGTAAGTGTCA		
Gene_0010-F	GGTAGACAGCAAGGATTCGC	358	GTP-binding protein
R	ACATTTGCTCTCCTCACGCT		

Supplementary Table S5. Primers for PCR to see sequence variations in *B. bassiana* isolates

Primers	Sequence (5'→3')	Tm (°C)	Amplicon size (bp)	Target gene
Gene_1250-F	GGCACCCATCTCTCCAGTAT	57	558	trypsin-like
R	ACGCAGGATTCTGTGCTTTC	58		protease
Gene_5653-F	TTCTCAGATTGGACGGCTTT	58	811	protein kinase
R	GAACTCCAAGCACCTTGTCC	58		
Gene_8961-F	GCTCTGGGCAGTACAAACGTA	58	946	chitinase-like
R	CAATGCTCATCACCGCTAGA	58		protein
Gene_9748-F	GCGATCTACGTGCTGAAATG	59	1,213	metalloprotease-
R	TGACAGACGGCATTACTCCA	60		like protein
Gene_15039-F	ATCATCCGGGTCAAATAGCA	60	1,501	eukaryotic
R	CCATCATTTGCGCTTCTTCT	60		aspartyl protease

(A) gDNA of *B. bassiana* JEF-007

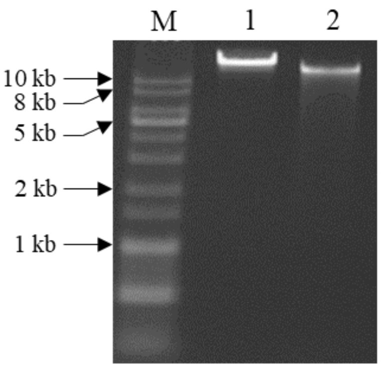

M, marker; 1, control DNA and 2, *B. bassiana* JEF-007 gDNA

(B) Statistics of filtered sub-reads

Mean sub-read length	Total No. of bases	N50	No. of reads
8,471	5,464,060,849	12,025	645,008

(C) Distribution of filtered sub-reads length

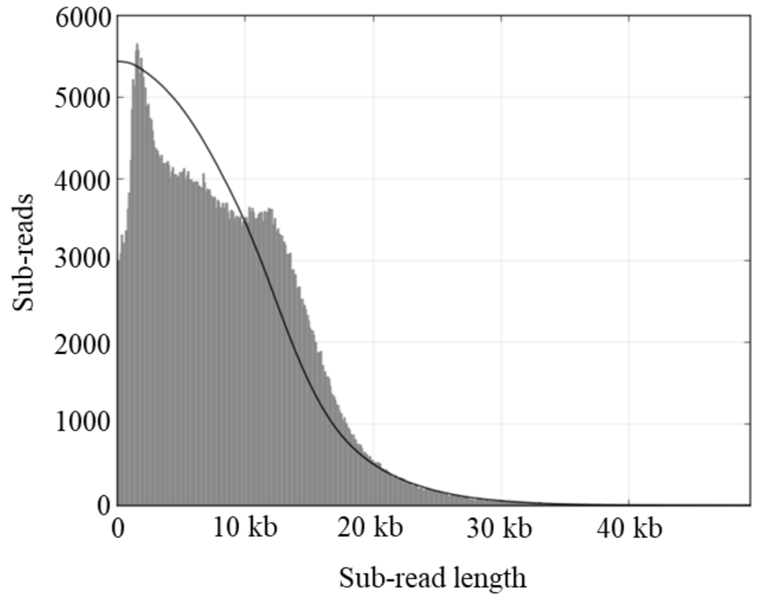

Supplementary Figure S1. Raw data for whole genome sequencing of B. bassiana JEF-007. Extraction of gDNA of JEF-007 (A), Statistics of filtered subreads (B); and Distribution of filtered subread lengths (C).

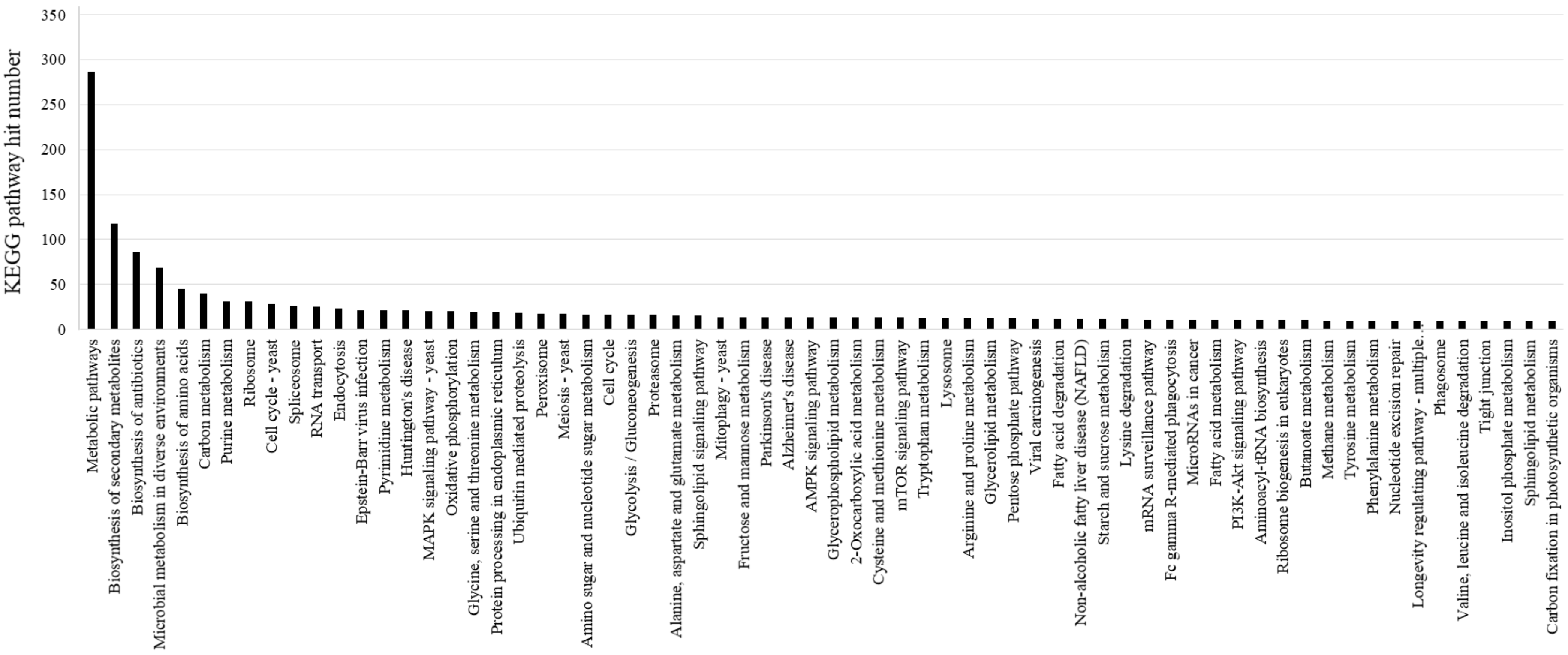

Supplementary Figure S2. KEGG analysis of predicted *B. bassiana* JEF-007 genes.

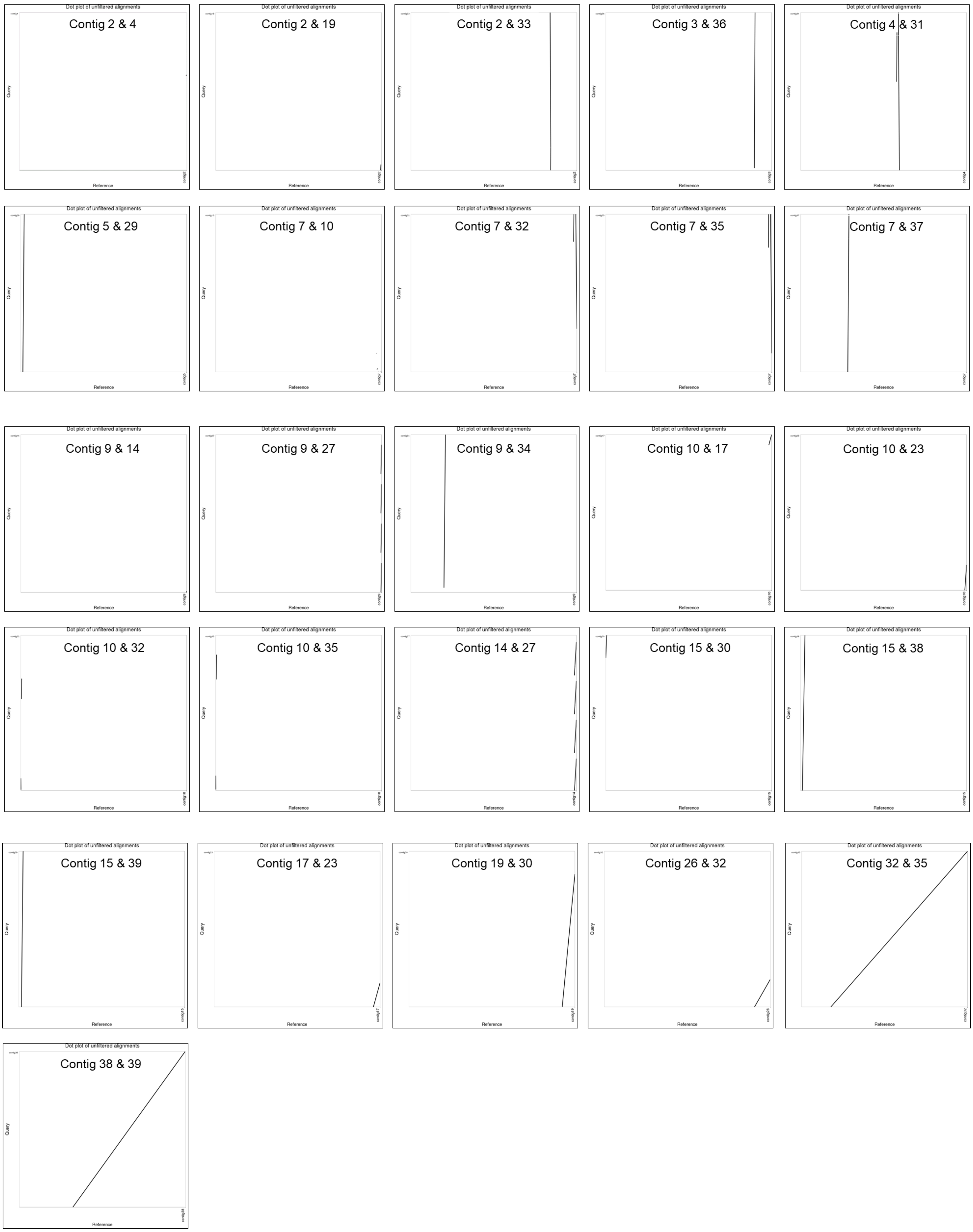

Supplementary Figure S3. Self-alignment of *B. bassiana* JEF-007 contigs to investigate duplication and repeats (Nummer program used). In the dot plots, reference and query were JEF-007 contigs, and in the following summarized table contig A and B came from JEF-007.

Self alignment of *B. bassiana* JEF-007 (Contig A and B came from the same JEF-007 genome)													
Contig A		Contig B		Aligned Length		Identity (%)	Total Length		**Alignment coverage (%)**		**Self aligned contig**	
start	end	start	end	Contig A	Contig B		Contig A	Contig B	**Contig A**	**Contig B**	**Contg A**	**Contig B**
4,288,934	4,297,954	1	9,022	9,021	9,022	100	4,313,377	246,642	0.21	3.66	contig2	contig19
3,618,414	3,635,915	20,327	2,911	17,502	17,417	99	4,313,377	20,327	0.41	85.68	contig2	contig33
3,632,818	3,635,764	1	2,901	2,947	2,901	98	4,313,377	20,327	0.07	14.27	contig2	contig33
4,296,841	4,313,377	2,122,613	2,139,348	16,537	16,736	97	4,313,377	3,539,716	0.38	0.47	contig2	contig4
3,331,321	3,348,120	236	17,020	16,800	16,785	100	3,721,617	17,020	0.45	98.62	contig3	contig36
2,049,010	2,055,693	9,314	2,671	6,684	6,644	96	3,539,716	21,250	0.19	31.27	contig4	contig31
2,085,747	2,107,148	2	21,250	21,402	21,249	99	3,539,716	21,250	0.6	100	contig4	contig31
39,366	64,474	1	25,046	25,109	25,046	100	3,115,233	25,046	0.81	100	contig5	contig29
2,284,344	2,286,095	188,207	186,456	1,752	1,752	99	2,356,961	1,591,247	0.07	0.11	contig7	contig10
2,292,012	2,293,948	24,735	26,668	1,937	1,934	97	2,356,961	1,591,247	0.08	0.12	contig7	contig10
2,293,948	2,301,276	26,868	34,236	7,329	7,369	97	2,356,961	1,591,247	0.31	0.46	contig7	contig10
2,312,983	2,314,532	374	1,924	1,550	1,551	99	2,356,961	1,591,247	0.07	0.1	contig7	contig10
2,341,821	2,343,370	1,924	374	1,550	1,551	99	2,356,961	1,591,247	0.07	0.1	contig7	contig10
2,354,089	2,356,846	6,256	9,013	2,758	2,758	100	2,356,961	1,591,247	0.12	0.17	contig7	contig10
2,310,992	2,314,649	17,362	21,017	3,658	3,656	100	2,356,961	21,017	0.16	17.4	contig7	contig32
2,341,704	2,356,961	21,017	5,756	15,258	15,262	100	2,356,961	21,017	0.65	72.62	contig7	contig32
2,310,992	2,314,649	13,649	17,304	3,658	3,656	100	2,356,961	17,304	0.16	21.13	contig7	contig35
2,341,704	2,356,961	17,304	2,050	15,258	15,255	100	2,356,961	17,304	0.65	88.16	contig7	contig35
670,194	686,799	5	16,589	16,606	16,585	100	2,356,961	16,589	0.7	99.98	contig7	contig37
1,982,306	1,987,760	677,945	672,491	5,455	5,455	100	1,987,760	677,945	0.27	0.8	contig9	contig14
1,980,638	1,987,760	9,617	2,495	7,123	7,123	100	1,987,760	38,659	0.36	18.43	contig9	contig27
1,980,638	1,987,760	19,281	12,160	7,123	7,122	100	1,987,760	38,659	0.36	18.42	contig9	contig27
1,980,638	1,987,760	28,946	21,824	7,123	7,123	100	1,987,760	38,659	0.36	18.43	contig9	contig27
1,980,638	1,987,760	38,611	31,489	7,123	7,123	100	1,987,760	38,659	0.36	18.43	contig9	contig27
397,111	414,917	544	18,324	17,807	17,781	100	1,987,760	18,324	0.9	97.04	contig9	contig34
1,564,476	1,590,428	364,455	390,418	25,953	25,964	100	1,591,247	390,418	1.63	6.65	contig10	contig17
1,574,789	1,591,247	100,976	84,507	16,459	16,470	100	1,591,247	100,976	1.03	16.31	contig10	contig23
374	1,924	19,352	20,900	1,551	1,549	99	1,591,247	21,017	0.1	7.37	contig10	contig32
6,256	9,013	8,628	5,871	2,758	2,758	100	1,591,247	21,017	0.17	13.12	contig10	contig32
374	1,924	15,640	17,187	1,551	1,548	99	1,591,247	17,304	0.1	8.95	contig10	contig35
6,256	9,013	4,922	2,165	2,758	2,758	100	1,591,247	17,304	0.17	15.94	contig10	contig35
669,852	672,139	9,355	11,642	2,288	2,288	100	677,945	38,659	0.34	5.92	contig14	contig27
669,852	672,139	19,019	21,306	2,288	2,288	100	677,945	38,659	0.34	5.92	contig14	contig27
669,852	672,139	28,684	30,971	2,288	2,288	100	677,945	38,659	0.34	5.92	contig14	contig27
670,164	672,139	1	1,976	1,976	1,976	100	677,945	38,659	0.29	5.11	contig14	contig27
671,799	677,945	1,824	7,949	6,147	6,126	99	677,945	38,659	0.91	15.85	contig14	contig27
671,799	677,945	11,490	17,613	6,147	6,124	99	677,945	38,659	0.91	15.84	contig14	contig27
671,799	677,945	21,154	27,278	6,147	6,125	99	677,945	38,659	0.91	15.84	contig14	contig27
671,799	677,945	30,819	36,943	6,147	6,125	99	677,945	38,659	0.91	15.84	contig14	contig27
1	3,201	3,210	10	3,201	3,201	100	561,348	22,032	0.57	14.53	contig15	contig30
6,099	14,671	1	8,408	8,573	8,408	98	561,348	8,409	1.53	99.99	contig15	contig38
8,871	14,671	1	5,750	5,801	5,750	99	561,348	5,751	1.03	99.98	contig15	contig39
374,770	390,418	100,976	85,327	15,649	15,650	100	390,418	100,976	4.01	15.5	contig17	contig23
227,856	246,642	22,032	3,211	18,787	18,822	100	246,642	22,032	7.62	85.43	contig19	contig30
35,198	38,876	1	3,704	3,679	3,704	99	38,876	21,017	9.46	17.62	contig26	contig32
3,706	21,017	1	17,304	17,312	17,304	100	21,017	17,304	82.37	100	contig32	contig35
2,715	8,409	1	5,751	5,695	5,751	97	8,409	5,751	67.73	100	contig38	contig39

Supplementary Figure S3. – *Continued (summary of the dot plots).*

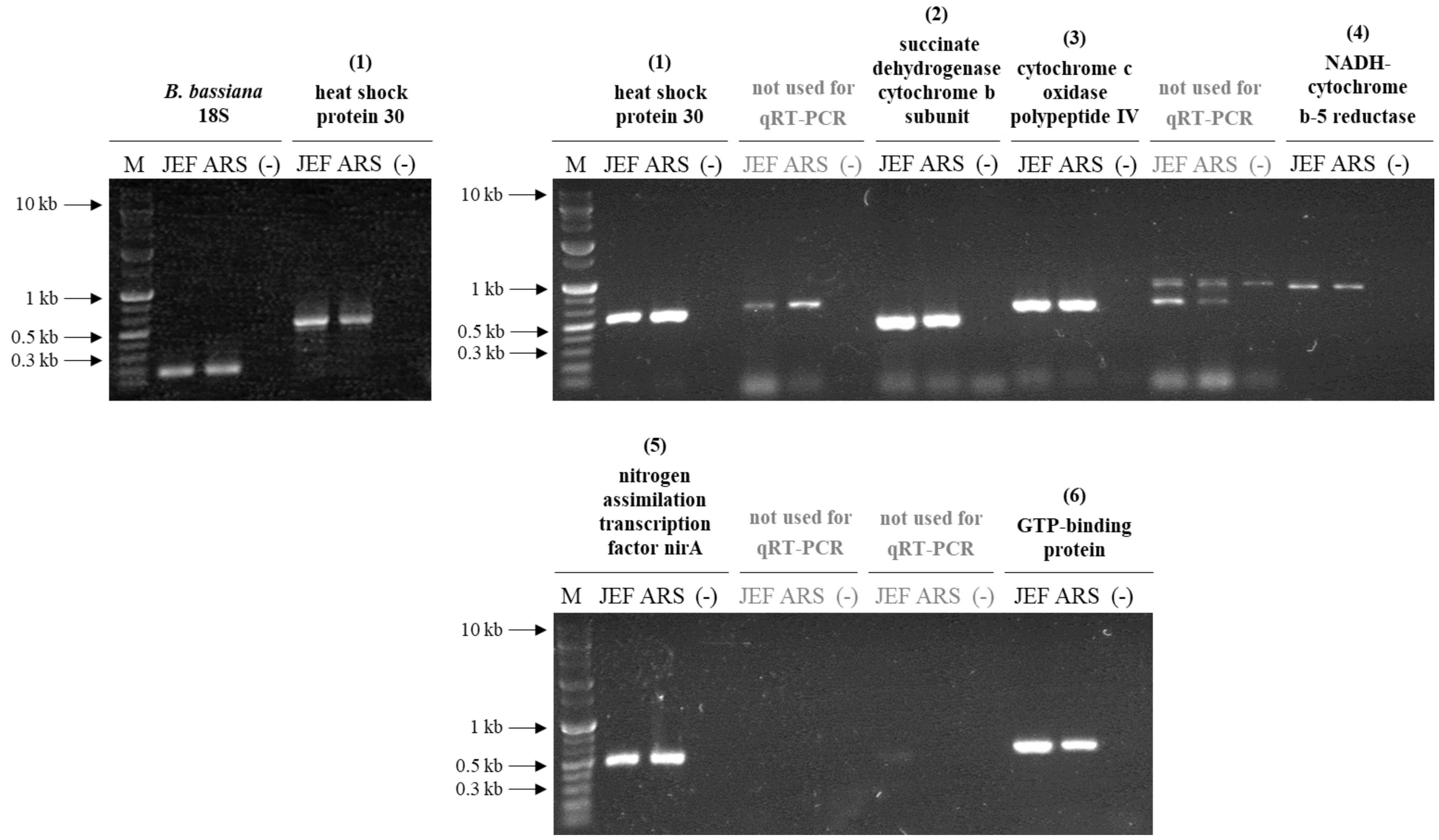

Supplementary Figure S4. Gels of reverse transcription (RT)-PCR products of JEF-007 and ARSEF2860 genes after electrophoresis in 0.8% agarose gel with 30 min running at 100 V. M, marker; JEF, JEF-007; ARS, ARSEF2860 and (-), negative control without template. Undetected or multiple-banded genes were not subjected for qRT-PCR analysis.

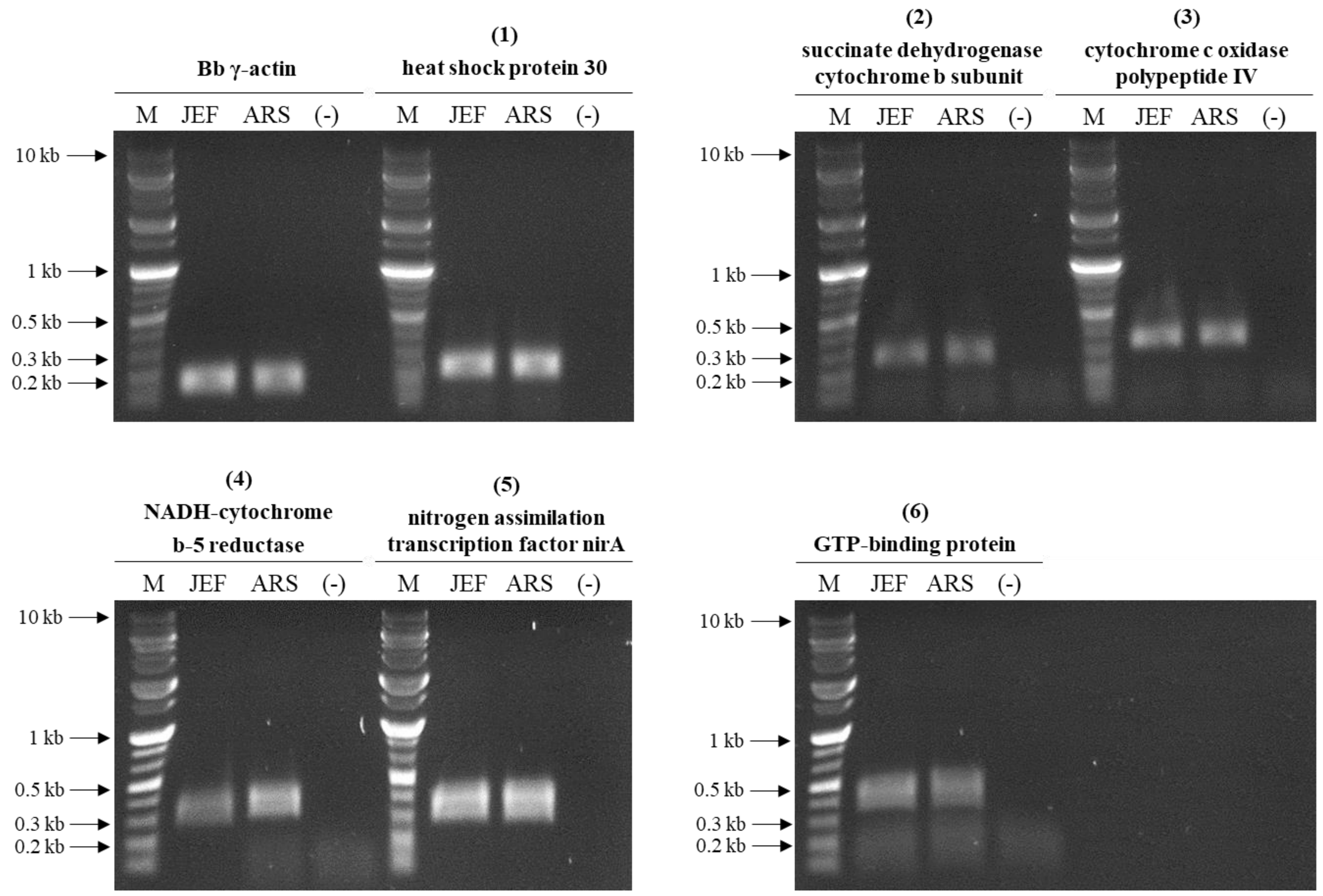

Supplementary Figure S5. Full-length gels of qRT-PCR products of JEF-007 and ARSEF2860 genes after electrophoresis in 0.8% agarose gel with 30 min running at 100 V. M, marker; JEF, JEF-007; ARS, ARSEF2860 and (-), negative control without template.
